# $\varepsilon '/\varepsilon$ in the Standard Model at the Dawn of the 2020s

**DOI:** 10.1140/epjc/s10052-020-8267-1

**Published:** 2020-08-06

**Authors:** Jason Aebischer, Christoph Bobeth, Andrzej J. Buras

**Affiliations:** 1 Department of Physics, University of California at San Diego, La Jolla, CA 92093 USA; 2 Physik Department, TU München, James-Franck-Straße, 85748 Garching, Germany; 3 TUM Institute for Advanced Study, Lichtenbergstr. 2a, 85748 Garching, Germany

## Abstract

We reanalyse the ratio $\varepsilon '/\varepsilon$ in the Standard Model (SM) using most recent hadronic matrix elements from the RBC-UKQCD collaboration in combination with most important NNLO QCD corrections to electroweak penguin contributions and the isospin-breaking corrections. We illustrate the importance of the latter by using their latest estimate from chiral perturbation theory (ChPT) based on the *octet* approximation for lowest-lying mesons and a very recent estimate in the *nonet* scheme that takes into account the contribution of $\eta _0$. We find $(\varepsilon '/\varepsilon )^{(8)}_\text {SM} = (17.4 \pm 6.1) \times 10^{-4}$ and $(\varepsilon '/\varepsilon )^{(9)}_\text {SM} = (13.9 \pm 5.2) \times 10^{-4}$, respectively. Despite a very good agreement with the measured value $(\varepsilon '/\varepsilon )_\text {exp} = (16.6 \pm 2.3) \times 10^{-4}$, the large error in $(\varepsilon '/\varepsilon )_\text {SM}$ still leaves room for significant new physics (BSM) contributions to this ratio. We update the 2018 master formula for $(\varepsilon '/\varepsilon )_\text {BSM}$ valid in any extension beyond the SM without additional light degrees of freedom. We provide new values of the penguin parameters $B_6^{(1/2)}(\mu )$ and $B_8^{(3/2)}(\mu )$ at the $\mu$-scales used by the RBC-UKQCD collaboration and at lower scales $\mathcal {O}(1\, \text {GeV})$ used by ChPT and Dual QCD (DQCD). We present semi-analytic formulae for $(\varepsilon '/\varepsilon )_\text {SM}$ in terms of these parameters and $\hat{\Omega }_\text {eff}$ that summarizes isospin-breaking corrections to this ratio. We stress the importance of lattice calculations of the $\mathcal {O}(\alpha _{\text {em}})$ contributions to the hadronic matrix elements necessary for the removal of renormalization scheme dependence at $\mathcal {O}(\alpha _{\text {em}})$ in the present analyses of $\varepsilon '/\varepsilon$.

## Introduction

The direct CP-violation in $K\rightarrow \pi \pi$ decays, represented by the ratio $\varepsilon '/\varepsilon$, plays a very important role in the tests of the Standard Model (SM) and more recently in constraining its possible extensions [1]. In the SM $\varepsilon '/\varepsilon$ is governed by QCD penguins (QCDP) but receives also an important contribution from the electroweak penguins (EWP), pointed out already in 1989 [2, 3], that entering $\varepsilon '/\varepsilon$ with the opposite sign to QCDP suppress this ratio significantly. The partial cancellation of these two contributions in addition to the evaluation of the hadronic matrix elements of QCDP and EWP operators is the reason why even today a precise prediction for $\varepsilon '/\varepsilon$ in the SM is not available. Yet, significant progress has been made during the last years and the goal of our paper is to update the SM value of $\varepsilon '/\varepsilon$ taking into account all available informations both from lattice QCD (LQCD) and analytic approaches most relevant for the evaluation of the Wilson coefficients but presently also for the estimate of the isospin-breaking corrections to the isospin amplitudes.

The situation of $\varepsilon '/\varepsilon$ in the SM before April 20, 2020 has been summarized by us in [4]. In short there are presently three approaches to calculate hadronic matrix elements entering $\varepsilon '/\varepsilon$:

- **Lattice QCD**, lead by the RBC-UKQCD LQCD collaboration. Using their results from 2015 for $K\rightarrow \pi \pi$ matrix elements [5, 6] and including isospin-breaking corrections from [7, 8] as done in [9, 10], leads to a value for $\varepsilon '/\varepsilon$ in the ballpark of $(1-2) \times 10^{-4}$. Although exhibiting a large error of $5 \times 10^{-4}$ the result lies one order of magnitude below the data. Taking these analyses at face value one could talk about an $\varepsilon '/\varepsilon$ anomaly of at most $3\,\sigma$.
- **DQCD approach** [11–13], which gave a support to these values and moreover provided an *upper bound* on $\varepsilon '/\varepsilon$ in the ballpark of $8\times 10^{-4}$. The main QCD dynamics suppressing $\varepsilon '/\varepsilon$ in this approach is represented by the meson evolution, which is necessary to match long-distance contributions to short-distance ones. On the other hand it has been argued in [13] that final state interactions (FSI) should have only a minor impact on $\varepsilon '/\varepsilon$ and the quoted bound does not include them.
- **Chiral Perturbation theory (ChPT)** [14–16] where, using ideas from ChPT, the authors found $\varepsilon '/\varepsilon = (14 \pm 5) \times 10^{-4}$ attributing an important role to FSI in this result. While in agreement with the measurement, the large uncertainty, that expresses the difficulties in matching long-distance and short-distance contributions in this framework, does not allow for any clear-cut conclusions.^1^

In view of the fact that LQCD calculations contain both the meson evolution^2^ and FSI, while the estimate of $\varepsilon '/\varepsilon$ in the other two approaches does not include one of them, we have recently proposed the optimal strategy for the evaluation of $\varepsilon '/\varepsilon$ as of 2020 [4, 19]

1. Use LQCD results for hadronic matrix elements of the dominant QCDP and EWP operators $Q_6$ and $Q_8$, respectively. They are represented by the parameters $B_6^{(1/2)}$ and $B_8^{(3/2)}$ defined in Sect. 2. On the other hand the hadronic matrix elements of $(V-A)\otimes (V-A)$ operators can be determined from the experimental data on the real parts of the $K\rightarrow \pi \pi$ amplitudes as performed in [9, 20]. In fact this procedure has been recently adopted with slight modifications by the RBC-UKQCD collaboration [21] with the goal to decrease their errors. This procedure is clearly legitimate when testing the consistency of the SM with the data, if the reduction of the uncertainties is significant. But the RBC-UKQCD analysis shows only a little gain and therefore we will not use it in the present work, but rather base the full analysis on *all* hadronic matrix elements from RBC-UKQCD that are absolutely free from new physics. We will only use the experimental values of ${{\,\mathrm{Re}\,}}A_{0,2}$ in the basic formula for $\varepsilon '/\varepsilon$ because they automatically take possible NP contributions into account.
2. Include isospin-breaking corrections from ChPT [15] that are compatible due to large uncertainties with the results obtained already 33 years ago in [22]. Very recently the latter analysis has been updated [23] and we will include these new findings as well.
3. Include NNLO QCD contributions to EWP in [24] thereby reducing the unphysical scale and renormalization scheme dependences in the matching at $\mu _W = \mathcal {O}(m_W)$, with the largest part due to the top-quark mass. The removal of the dependence on $\mu _c$ at NNLO has still to be done, see also the next point.
4. Take into account NNLO QCD contributions to QCDP [25, 26]. This reduces the left-over renormalization scale uncertainties present at the NLO level, in particular those due to the matching scale $\mu _c$.

Recently significant progress in the estimate of $\varepsilon '/\varepsilon$ in the SM has been made through the improved values of the $K\rightarrow \pi \pi$ hadronic matrix elements presented by the RBC-UKQCD collaboration [21]. Not only statistical errors have been significantly decreased but also a better agreement with the experimental values of the $\pi \pi$ strong interaction phases $\delta _{0,2}$ has been obtained. The RBC-UKQCD collaboration, using their new results for the hadronic matrix elements and known Wilson coefficients at the NLO level [20, 27–31] but not accounting for isospin-breaking corrections, finds [21]

1 $$\begin{aligned} (\varepsilon '/\varepsilon )_\text {SM}&= (21.7 \pm 8.4) \times 10^{-4},\quad (\text {RBC-UKQCD}-2020) \end{aligned}$$

to be compared with the experimental world average from NA48 [32] and KTeV [33, 34] collaborations,

2 $$\begin{aligned} (\varepsilon '/\varepsilon )_\text {exp}&= (16.6 \pm 2.3) \times 10^{-4}. \end{aligned}$$

While the result in (1) is in full agreement with the experimental value in (2) the theoretical error of $39\%$ does not allow for clear cut conclusions whether some amount of new physics contributions is present in $\varepsilon '/\varepsilon$ or not. The same is the case of the earlier updated ChPT analysis [15], which resulted in

3 $$\begin{aligned} (\varepsilon '/\varepsilon )_\text {SM}&= (14 \pm 5) \times 10^{-4} \,,\qquad (\text {ChPT}-2019), \end{aligned}$$

with an error of 36%, very close to the LQCD one. But it should be remarked that with the present best values of the CKM parameters as used by us the central value in (3) would be raised to $15.0 \times 10^{-4}$.

Despite large errors both results deviate significantly from the DQCD values of $\varepsilon '/\varepsilon$ in the ballpark of $5\times 10^{-4}$ stressed in particular in [17]. While there is no question about that meson evolution necessary for a proper matching between Wilson coefficients and hadronic matrix elements at scales $\mathcal {O}(1\, \text {GeV})$ must play a role in the evaluation of $\varepsilon '/\varepsilon$ it appears from present RBC-UKQCD results that precisely in the case of the matrix element of the $Q_6$ operator its suppression is overcompensated by other QCD dynamics which was hidden due to the contamination of the excited $\pi \pi$ states present in their 2015 analysis. It has been removed in the latest analysis. In fact as we will see soon the value of $\varepsilon '/\varepsilon$ obtained using the optimal procedure with hadronic matrix elements from [21], agrees very well with the one advocated in [15] and given in (3). Yet, it is not evident at present that FSI, as claimed by ChPT experts, are responsible for this agreement. Possibly other dynamical QCD effects apparently not taken into account both in the ChPT and DQCD approaches are responsible for the enhancement of $\varepsilon '/\varepsilon$ relative to DQCD expectations.^3^ However, a clear-cut conclusion on this issue is difficult because of rather different techniques that are used in these three approaches. The fact that the central value in (3) differs significantly from the central LQCD value in (1) is dominantly due to the omission of isospin-breaking effects in the RBC-UKQCD prediction that are included in (3).

Even if the new improved calculation of $K\rightarrow \pi \pi$ hadronic matrix elements in [21] is an important advance towards the accurate calculation of $\varepsilon '/\varepsilon$, the result in (1) does not represent the present SM value of $\varepsilon '/\varepsilon$ properly. Indeed, as we emphasized in [4] the hadronic matrix elements in question are only a part of the $\varepsilon '/\varepsilon$ story. The three additional advances, listed in the context of the optimal strategy, that are not taken into account in the result in (1) are also important, in particular because they all lower the value of $\varepsilon '/\varepsilon$. As we will demonstrate below, the final result for $\varepsilon '/\varepsilon$ differs significantly from the one obtained by the RBC-UKQCD collaboration. Indeed after including isospin-breaking effects from [15] that include the effects from the *octet* of lowest-lying mesons and NNLO QCD corrections to EWP contributions, we find using the hadronic matrix elements of RBC-UKQCD

4 $$\begin{aligned} (\varepsilon '/\varepsilon )^{(8)}_\text {SM} = (17.4 \pm 6.1) \times 10^{-4} \,. \end{aligned}$$

On the other hand including the singlet $\eta _0$ in this estimate one arrives at [23]

5 $$\begin{aligned} \boxed {(\varepsilon '/\varepsilon )^{(9)}_\text {SM} = (13.9 \pm 5.2) \times 10^{-4}} \,. \end{aligned}$$

Both results agree very well with experiment and with the ChPT expectations but in view of our comments on the ChPT analysis are on a more solid footing. We expect further reduction of $\varepsilon '/\varepsilon$ by roughly (5–10)% when NNLO QCD corrections to QCD penguin contributions will be taken into account [25, 26]. We look forward to the final results of these authors.

Our paper is organized as follows. In Sect. 2, after recalling a number of basic formulae, we determine the parameters $B_6^{(1/2)}$ and $B_8^{(3/2)}$ using the recent RBC-UKQCD results and compare them with the expectations from ChPT [15] and DQCD [12, 13]. It turns out that while there is a good agreement on the value of $B_6^{(1/2)}$ between LQCD and ChPT, the rather precise value of $B_8^{(3/2)}$ from RBC-UKQCD is by a factor of 1.5 larger than the ChPT one when both are evaluated at $\mu = 1\,\text {GeV}$. On the contrary, while there is a good agreement on the value of $B_8^{(3/2)}$ between LQCD and DQCD [12], the most recent value of $B_6^{(1/2)}$ from RBC-UKQCD is by a factor of two larger than the values quoted in [12, 13]. We close this section with an updated formula for $\varepsilon '/\varepsilon$ in terms of $B_6^{(1/2)}$ and $B_8^{(3/2)}$. In Sect. 3 we derive the results in (4) and (5) which take into account the updated isospin-breaking effects [15, 23] and also NNLO QCD corrections to EWP contributions [24]. We also perform a detailed anatomy of various contributions. In Sect. 4 we update the BSM master formula for $\varepsilon '/\varepsilon$ [35, 36] in view of the new RBC-UKQCD results. A brief summary and an outlook are given in Sect. 5. Some additional information on the numerical analysis are given in appendices. This includes the values of the hadronic matrix elements from RBC-UKQCD and the Wilson coefficients at various scales. We discuss in detail the effect of isospin-breaking corrections present in the renormalization group (RG) flow on $\varepsilon '/\varepsilon$ in Appendix C.

## Basic formulae

### Preliminaries

The amplitudes for $K^0\rightarrow (\pi \pi )_I$, with $I=0,2$ denoting strong isospin of the final state, are given as

6 $$\begin{aligned} A_0&= \mathcal {N}_{\Delta S = 1} \sum _{i=1}^{10} \big [z_i(\mu ) + \tau y_i(\mu ) \big ] \langle Q_i(\mu )\rangle _0 \,, \end{aligned}$$

7 $$\begin{aligned} A_2&= \mathcal {N}_{\Delta S = 1} \sum _{i=1}^{10} \big [ z_i(\mu ) + \tau y_i(\mu )\big ] \langle Q_i(\mu )\rangle _2 \,, \end{aligned}$$

where $z_i(\mu )$ and $y_i(\mu )$ are the $\Delta S = 1$ Wilson coefficients and $\langle Q_i(\mu )\rangle _{0,2}$ the hadronic matrix elements of the operators $Q_i$, both in the $\overline{\text {MS}}$ scheme at the low-energy factorization scale $\mu$ in the $N_f = 3$ flavour theory [20]. By convention the strong phase shifts $\delta _{0,2}$ are not included in $A_{0,2}$, and therefore the $\langle Q_i(\mu )\rangle _{0,2}$ are real-valued. Further

8 $$\begin{aligned} \mathcal {N}_{\Delta S = 1} = \frac{G_F}{\sqrt{2}} V_{us}^* V_{ud}^{} , \qquad \tau = - \frac{V_{ts}^*\, V_{td}^{}}{V_{us}^* V_{ud}^{}}. \end{aligned}$$

The real parts ${{\,\mathrm{Re}\,}}A_{0,2}$ are given entirely by the $z_i$, because the $y_i$ are strongly suppressed by $\tau \sim \mathcal {O}(10^{-3})$, on the other hand the imaginary parts ${{\,\mathrm{Im}\,}}A_{0,2} \propto {{\,\mathrm{Im}\,}}(V_{ts}^*\, V_{td}^{})$ and depend only on $y_i$. The Wilson coefficients of the QCD penguin (QCDP) operators $i=3,\ldots ,6$ are usually larger compared to those of the electroweak penguin (EWP) operators $i=7,\ldots ,10$, as can be seen in Table 7.

The scheme and scale dependences cancel between the Wilson coefficients and the matrix elements individually in $A_0$ and $A_2$. We will take advantage of this freedom to use different scales $\mu _0$ and $\mu _2$ in the evaluation of $A_0$ and $A_2$, respectively. In particular we choose the values at which the RBC-UKQCD lattice collaboration presents their results of the $I=0$ [21] and $I=2$ [6] matrix elements. There are only seven linearly independent $\langle Q_i(\mu ) \rangle _0$ and three linearly independent $\langle Q_i(\mu )\rangle _2$ in the $N_f = 3$ flavour theory [9, 20], since RBC-UKQCD work in the isospin-symmetric limit, where also QED corrections are not included yet.

We remind that the amplitudes $A_{0,2}$ and the strong phase shifts $\delta _{0,2}$ are related to the decay amplitudes relevant for $\varepsilon '/\varepsilon$ as follows

9 $$\begin{aligned} \begin{aligned} A(K^0 \rightarrow \pi ^+\pi ^-)&= \frac{1}{h} \Big [ A_0 e^{i \delta _0} + \frac{1}{\sqrt{2}} A_2 e^{i \delta _2} \Big ] ,\\ A(K^0 \rightarrow \pi ^0\pi ^0)&= \frac{1}{h} \Big [ A_0 e^{i \delta _0} - \sqrt{2}\, A_2 e^{i \delta _2} \Big ] ,&\end{aligned} \end{aligned}$$

with the experimental values of $A_{0,2}$ for $h = 1$ given in Table 2, whereas RBC-UKQCD works with the convention $h = \sqrt{3/2}$. These relations are valid also in the presence of finite QED corrections, as long as virtual infrared-divergent contributions, and also Coulomb corrections, are properly subtracted and combined with real photon radiation [8] when determining the amplitudes and phases from data.

### Basic formula for $\varepsilon '/\varepsilon$

As in [9], our starting expression is the formula

10 $$\begin{aligned} \frac{\varepsilon '}{\varepsilon }&= -\,\frac{\omega _+}{\sqrt{2}\,|\varepsilon _K|} \left[ \, \frac{{{\,\mathrm{Im}\,}}\widetilde{A}_0}{{{\,\mathrm{Re}\,}}A_0}\, (1 - \hat{\Omega }_\text {eff}) - \frac{1}{a} \, \frac{{{\,\mathrm{Im}\,}}A_2}{{{\,\mathrm{Re}\,}}A_2} \,\right] , \end{aligned}$$

where [8, 15]

11 $$\begin{aligned} \omega _+&= a\,\frac{{{\,\mathrm{Re}\,}}A_2}{{{\,\mathrm{Re}\,}}A_0} = (4.53 \pm 0.02) \times 10^{-2} ,&a&= 1.017 . \end{aligned}$$

Here *a* and $\hat{\Omega }_\text {eff}$ summarise isospin-breaking corrections. The latter include strong isospin violation $(m_u \ne m_d)$, the correction to the isospin limit coming from $\Delta I=5/2$ transitions and electromagnetic corrections as first summarized in [7, 8] and recently updated in [15]

12 $$\begin{aligned} \hat{\Omega }_\text {eff}&= \hat{\Omega }_\text {eff}^{(8)} = (17.0 \pm 9.1) \times 10^{-2} . \end{aligned}$$

These analyses are based on the so-called *octet scheme* which includes only the octet of the lowest-lying pseudoscalar mesons. The inclusion of the singlet $\eta _0$ in the *nonet scheme* has been known already for 33 years [22, 37] to give stronger suppression of $\varepsilon '/\varepsilon$ through the $\eta -\eta ^\prime$ mixing, but only very recently this estimate has been updated and put on a more solid basis than it was possible in 1987. With

13 $$\begin{aligned} \hat{\Omega }_\text {eff}&= \hat{\Omega }_\text {eff}^{(9)} = (29 \pm 7) \times 10^{-2} , \end{aligned}$$

the role of isospin-breaking effects is enhanced relative to the ChPT estimate in (12).

The inclusion of the isospin-breaking corrections requires a modification in the evaluation of the ${{\,\mathrm{Im}\,}}A_0$ part in $\varepsilon '/\varepsilon$ as follows [9]

14 $$\begin{aligned}&{{\,\mathrm{Im}\,}}A_0 \quad \rightarrow \quad {{\,\mathrm{Im}\,}}\widetilde{A}_0 = \mathcal {N}_{\Delta S = 1} {{\,\mathrm{Im}\,}}\tau \nonumber \\&\quad \times \left[ \sum _{i=3}^{6} y_i(\mu ) \langle Q_i(\mu )\rangle _0 + \sum _{i=7}^{10} \frac{y_i(\mu ) \langle Q_i(\mu )\rangle _0}{a (1 - \hat{\Omega }_\text {eff})}\right] , \end{aligned}$$

such that only leading isospin-breaking corrections are included.

A strong reduction of the uncertainty of $\varepsilon '/\varepsilon$ can be achieved firstly [20] by the use of the experimental values of ${{\,\mathrm{Re}\,}}A_{0,2}$ in the denominators of (10). Secondly, the real parts of the relations (6) and (7) allow to eliminate one $\langle Q_j(\mu _0)\rangle _0$ and one $\langle Q_k(\mu _2)\rangle _2$, respectively, in favour of the measured values of ${{\,\mathrm{Re}\,}}A_0$ and ${{\,\mathrm{Re}\,}}A_2$, respectively. These can then be used in the numerators ${{\,\mathrm{Im}\,}}\widetilde{A}_0$ and ${{\,\mathrm{Im}\,}}A_2$, as proposed in [9]. The particular choice of *j* and *k* is subject to optimisation. However, as already announced previously we will not use this procedure here.

The real parts of the isospin amplitudes $A_{0,2}$ in (10) are then extracted from the branching ratios on $K\rightarrow \pi \pi$ decays in the isospin limit. In the limit $a = 1$ and $\hat{\Omega }_\text {eff}= 0$ the formula in (10) reduces to the one used by RBC-UKQCD [21], where all isospin breaking-corrections except for EWP contributions at the NLO level have been set to zero.

### Extracting $B_6^{(1/2)}$ and $B_8^{(3/2)}$ from LQCD

In the past the so-called bag factors have been frequently used in phenomenological analyses and it is interesting to provide their values in view of the updated $I=0$ matrix elements. The $B_6^{(1/2)}$ and $B_8^{(3/2)}$ parameters, that enter the formula (20), are defined as follows

15 $$\begin{aligned} \langle Q_6(\mu ) \rangle _0&= -\,4 h \left[ \frac{m_K^2}{m_s(\mu ) + m_d(\mu )}\right] ^2 (F_K - F_\pi ) \,B_6^{(1/2)}, \end{aligned}$$

16 $$\begin{aligned} \langle Q_8(\mu ) \rangle _2&= \sqrt{2} h \left[ \frac{m_K^2}{m_s(\mu ) + m_d(\mu )} \right] ^2 F_\pi \,B_8^{(3/2)}, \end{aligned}$$

with [22, 38]

17 $$\begin{aligned} B_6^{(1/2)}&= B_8^{(3/2)}= 1 \end{aligned}$$

in the large-*N* limit. We have introduced the factor *h* in order to emphasize different normalizations of these matrix elements present in the literature.

We find from the latest RBC-UKQCD results for $I=0$ [21] matrix elements at the scales $\mu = 1\,\text {GeV}$, $\mu = \mu _c = 1.3\,\text {GeV}$ and $\mu = \mu _0 = 4.006\,\text {GeV}$

18 $$\begin{aligned} \begin{aligned} B_6^{(1/2)}(1.0\,\text {GeV})&= 1.49 \pm 0.11|_\text {stat} \pm 0.23 |_\text {syst} = 1.49 \pm 0.25,\\ B_6^{(1/2)}(\mu _c)&= 1.36 \pm 0.10|_\text {stat} \pm 0.21 |_\text {syst} = 1.36 \pm 0.23,\\ B_6^{(1/2)}(\mu _0)&= 1.11 \pm 0.08|_\text {stat} \pm 0.18 |_\text {syst} = 1.11 \pm 0.20, \end{aligned} \end{aligned}$$

and for $I=2$ from [6] for $\mu =1\,\text {GeV}$, $\mu _c = 1.3 \,\text {GeV}$ and $\mu _2 = 3.0 \,\text {GeV}$

19 $$\begin{aligned} \begin{aligned} B_8^{(3/2)}(1.0\,\text {GeV})&= 0.85 \pm 0.02|_\text {stat} \pm 0.05 |_\text {syst} = 0.85 \pm 0.05,\\ B_8^{(3/2)}(\mu _c)&= 0.79 \pm 0.02|_\text {stat} \pm 0.05 |_\text {syst} = 0.79 \pm 0.05,\\ B_8^{(3/2)}(\mu _2)&= 0.70 \pm 0.02|_\text {stat} \pm 0.04 |_\text {syst} = 0.70 \pm 0.04, \end{aligned} \end{aligned}$$

to be compared with the 2015 values $B_6^{(1/2)}(\mu _c) = 0.57 \pm 0.19$ and $B_8^{(3/2)}(\mu _c) = 0.76 \pm 0.05$ from RBC-UKQCD [5, 6]. In principle only^4^ the central value of $B_6^{(1/2)}$ has been changed by a factor of more than two, but with slightly larger uncertainty, which would correspond to a $2.6\,\sigma$ discrepancy. However, in view that the systematic uncertainty of the 2015 results for the $I=0$ matrix elements has been underestimated [21], the uncertainty quoted for the 2015 result of $B_6^{(1/2)}(\mu _c)$ must not be taken at face value anymore.

The new value of $B_6^{(1/2)}$ is in the ballpark of values advocated in [15], but it is unclear to us at present whether this is a numerical coincidence or due to FSI dynamics. Moreover, the large uncertainty in the value of $B_6^{(1/2)}$ does not yet rule out the values of $B_6^{(1/2)}< 1.0$ as expected from the DQCD approach [12]. Similar, the decrease of both parameters with increased $\mu$, pointed out already in [20] and seen above, is also present below $1\,\text {GeV}$ within the DQCD allowing smooth matching between hadronic matrix elements and Wilson coefficients. On the other hand it turns out that while there is a good agreement on the value of $B_8^{(3/2)}$ between LQCD and DQCD [12], its rather precise value from RBC-UKQCD is by a factor of 1.5 larger than the ChPT one, in the ballpark of 0.55, when both are evaluated at $\mu = 1\,\text {GeV}$.

**Table 1:** Coefficients entering the semi-analytic formula (20), when the amplitudes $A_0$ and $A_2$ are evaluated at the scales $\mu _0$ and $\mu _2$, respectively, for different choices of $(\mu _0, \mu _2)$. The central values for $(\varepsilon '/\varepsilon )^{(8,9)}$ with these approximate formulas are given in the last two lines.

$(\mu _0,\,\mu _2)$[GeV]	$(1.0, \, 1.0)$	$(1.3,\,1.3)$	$(4.006,\, 3.0)$
$B_6^{(1/2)}(\mu _0)$	$1.49 \pm 0.25$	$1.36 \pm 0.23$	$1.11 \pm 0.20$
$B_8^{(3/2)}(\mu _2)$	$0.85 \pm 0.05$	$0.79 \pm 0.05$	$0.70 \pm 0.04$
$m_d\,$[MeV]	6.37	5.52	$(3.88,\, 4.16)$
$m_s\,$[MeV]	125.48	108.81	$(76.50,\, 81.89)$
$a^\text {QCDP}(\mu _0)$	$-\,2.86$	$-\,3.37$	$-\,5.64$
$a_6^{(1/2)}(\mu _0)$	15.15	16.98	22.77
$a^\text {EWP}(\mu _0, \mu _2)$	$-\,2.02$	$-\,2.12$	$-\,2.27$
$a_8^{(3/2)}(\mu _2)$	8.00	8.79	9.85
$10^4 \times (\varepsilon '/\varepsilon )^{(8)}$	17.2	17.1	17.3
$10^4 \times (\varepsilon '/\varepsilon )^{(9)}$	13.7	13.7	13.9

### An analytic formula for $\varepsilon '/\varepsilon$

As is well-known and shown also in the full analysis later, $\varepsilon '/\varepsilon$ is strongly dominated by the two terms $\propto \langle Q_6 \rangle _0 \sim B_6^{(1/2)}$ and $\propto \langle Q_8 \rangle _2 \sim B_8^{(3/2)}$. For convenience we provide a semi-analytic result of $\varepsilon '/\varepsilon$ in terms of these two parameters. Contrary to [4, 9], we evaluate $A_0$ and $A_2$ at the two different scales $\mu _0$ and $\mu _2$ and use now for the remaining matrix elements the RBC-UKQCD results. Then

20 $$\begin{aligned} \frac{\varepsilon '}{\varepsilon }&= {{\,\mathrm{Im}\,}}\lambda _t \cdot \left[ a (1 - \hat{\Omega }_\text {eff}) \left( a^\text {QCDP} + a_6^{(1/2)} B_6^{(1/2)}\right) \nonumber \right. \\&\quad \left. - a^\text {EWP} - a_8^{(3/2)} B_8^{(3/2)}\right] \,, \end{aligned}$$

with the coefficients given in Table 1 for various choices of $(\mu _0,\, \mu _2)$. The numerical input of the various parameters entering (20) is given in Table 2 and details on the Wilson coefficients at scales $\mu _{0,2}$ are collected in Appendix B. The quark masses in (15) and (16) have been calculated as well at the two scales $\mu _0$ and $\mu _2$, respectively. The coefficients $a_i^j$ are comparable to [4], but differ because of the updated values for the remaining $I=0$ matrix elements and changed values of the down- and strange-quark masses. Note that $a^\text {EWP}$ contains the $I=0$ and $I=2$ contributions of the EWPs at the scales $(\mu _0,\, \mu _2) = (4.006,\, 3.0)\,\text {GeV}$, where the QCDP matrix elements for $I=2$ are zero because the lattice calculation is done in the isospin limit. In general, when using the RG equations to evolve these matrix elements to different scales $\mu _{0,2}$, the isospin-breaking in quark charges in the RG flow lead to nonvanishing $I=2$ QCDP matrix elements that would also contribute to $a^\text {EWP}$. As explained in more detail in Appendix C, we evolve the matrix elements of the operators from the initial scales $\mu _{0,2}$ to the scales $\mu = 1.3\,\text {GeV}$ and $1.0\,\text {GeV}$ only with NLO QCD RG equations instead of NLO QCD$\,\times \,$QED, which maintains isospin relations for these matrix elements.

## $\varepsilon '/\varepsilon$ in the Standard Model

**Table 2:** Numerical input: The CKM elements and combinations thereof and the uncertainties are derived from Wolfenstein parameters from PDG 2019. The experimental results for $K\rightarrow \pi \pi$ amplitudes ${{\,\mathrm{Re}\,}}A_{0,2}|_\text {exp}$ are for normalization $h=1$. The $\overline{\text {MS}}$ quark masses are FLAG averages for $N_f = 2 + 1$ from [42–47].

Parameter	Value	References	Parameter	Value	References
$G_F$	$1.166379 \times 10^{-5} \,\text {GeV}^{-2}$	[39]			
$\lambda$	0.22453(44)	[39]	*A*	0.836(15)	[39]
${\overline{\rho }}$	$0.122(^{+18}_{-17})$	[39]	${\overline{\eta }}$	$0.355(^{+12}_{-11})$	[39]
$V_{ud}$	0.97446(10)		$V_{td}^{} V_{ts}^*$	$[-3.40(15) + i\, 1.45(8)]\times 10^{-4}$	
$V_{us}$	0.22453(45)		$\tau$	$[\,15.58(67) - i\, 6.62(35)]\times 10^{-4}$	
${{\,\mathrm{Re}\,}}A_0|_\text {exp}$	$27.04(1) \times 10^{-8}$ GeV	[40]	$\varepsilon _K$	0.002228(11)	[39]
${{\,\mathrm{Re}\,}}A_2|_\text {exp}$	$1.210(2) \times 10^{-8}$ GeV	[40]	$m_K$	497.614 MeV	[39]
$F_\pi$	130.41(20) MeV	[39]	$m_d(2\,\text {GeV})$	4.67(9) MeV	[41]
$F_K/F_\pi$	1.194(5)	[41]	$m_s(2\,\text {GeV})$	92.0(1.1) MeV	[41]

The new results for the $I=0$ matrix elements from RBC-UKQCD imply a modification of $\varepsilon '/\varepsilon$ in the SM relative to those values presented in 2015 in [5, 9, 10], taking into account additional advances listed in Sect. 1. Here we include the isospin-breaking corrections $\hat{\Omega }_\text {eff}$ and NNLO QCD corrections to EWPs calculated in [24]. Both contributions lead to a considerable reduction of $\varepsilon '/\varepsilon$, as discussed previously [4]. Note that the RBC-UKQCD collaboration [21] prefers to use the magnitude of the isospin-breaking corrections from ChPT in the octet scheme (12) exclusively as an estimate of their size, thereby introducing an additional large uncertainty in $\varepsilon '/\varepsilon$. In contrast to previous predictions [4, 9], here we use in obtaining the final result for $\varepsilon '/\varepsilon$ directly the LQCD values of matrix elements $\langle Q_i(\mu _0) \rangle _0$ and $\langle Q_i(\mu _2) \rangle _2$. For the interested readers, we provided the updated values of the two most important bag factors $B_6^{(1/2)}$ and $B_8^{(3/2)}$ in Sect. 2.3.

**Fig. 1:**
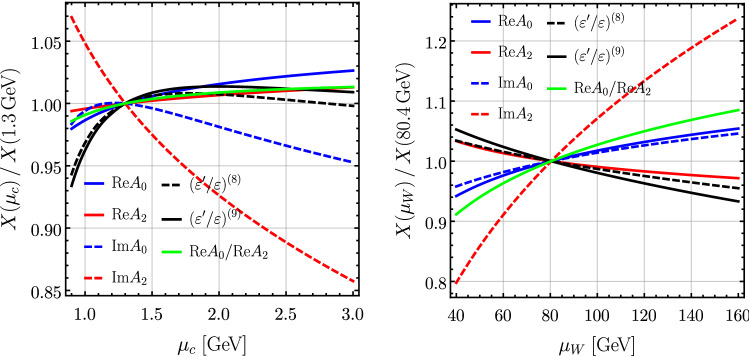
The $\mu _c$ dependence [left] and $\mu _W$ dependence [right] at NLO accuracy of the various quantities normalized to their value at $\mu _c = 1.3\,\text {GeV}$ and $\mu _W = 80.4\,\text {GeV}$, respectively

We find for the amplitudes ($h=1$)

21 $$\begin{aligned} {{\,\mathrm{Re}\,}}A_0&= \left( 24.63 \pm 2.65 \big |^\text {ME}_\text {stat} \pm 3.87 \big |^\text {ME}_\text {syst} \right. \nonumber \\&\quad \left. {}^{+0.63}_{-0.33} \big |_{\mu _c} \; {}^{+1.08}_{-0.97} \big |_{\mu _W} \right) 10^{-8} \,\text {GeV}, \end{aligned}$$

22 $$\begin{aligned} {{\,\mathrm{Re}\,}}A_2&= \left( \;1.23\, \pm 0.03 \big |^\text {ME}_\text {stat} \pm 0.07 \big |^\text {ME}_\text {syst} \right. \nonumber \\&\quad \left. {}^{+0.02}_{-0.01} \big |_{\mu _c} \; {}^{+0.03}_{-0.03} \big |_{\mu _W} \right) 10^{-8} \,\text {GeV}, \end{aligned}$$

and

23 $$\begin{aligned} {{\,\mathrm{Im}\,}}A_0&= \left( -5.74 \pm 0.53 \big |^\text {ME}_\text {stat} \pm 0.90 \big |^\text {ME}_\text {syst} \pm 0.30 \big |_\text {CKM} \; \right. \nonumber \\&\quad \left. {}^{+0.00}_{-0.26}\big |_{\mu _c} \; {}^{+0.21}_{-0.17} \big |_{\mu _W} \pm 0.01 \big |_{m_t} \right) 10^{-11} \,\text {GeV}, \end{aligned}$$

24 $$\begin{aligned} {{\,\mathrm{Im}\,}}A_2&= \left( -7.09 \pm 0.23 \big |^\text {ME}_\text {stat} \pm 0.43 \big |^\text {ME}_\text {syst} \pm 0.37 \big |_\text {CKM} \; \right. \nonumber \\&\quad \left. {}^{+0.34}_{-1.01}\big |_{\mu _c} \; {}^{+1.34}_{-1.00} \big |_{\mu _W} \pm 0.12 \big |_{m_t} \right) 10^{-13} \,\text {GeV}, \end{aligned}$$

where NNLO QCD corrections have been included in EWP parts [4]. The statistical uncertainties due to the matrix elements (ME, stat) were determined including the available correlations for $I=0$, whereas the systematic ones (ME, syst) are based on the overall $15.7\,\%$ for $I=0$ and $(3-6)\,\%$ for $I=2$, as estimated by RBC-UKQCD in [6, 21], respectively. For comparison, these values are very close to the RBC-UKQCD predictions ${{\,\mathrm{Re}\,}}A_0 = 24.44 \times 10^{-8} \,\text {GeV}$, ${{\,\mathrm{Re}\,}}A_2 = 1.22 \times 10^{-8} \,\text {GeV}$, ${{\,\mathrm{Im}\,}}A_0 = -5.70 \times 10^{-11} \,\text {GeV}$, ${{\,\mathrm{Im}\,}}A_2 = -6.81 \times 10^{-13} \,\text {GeV}$, from Eqs.  (77a, 85, 90) [21] and Eq.  (64) [6], respectively.^5^ The scale uncertainties are obtained by varying $\mu _c\in [1.0,\,3.0]\,\text {GeV}$ and $\mu _W\in [50,\,140]\,\text {GeV}$ for the NLO expressions, shown in Fig. 1. Note that we use $m_t(\mu _W)$, and hence the $\mu _W$ variation includes the top-mass scheme dependence. We emphasize that the $\mu _W$ uncertainty for ${{\,\mathrm{Im}\,}}A_{0,2}$, and $\varepsilon '/\varepsilon$, is very conservative, because we actually include here partial NNLO QCD corrections to EWPs [24], which remove the implicit $\mu _W$ dependence associated with the top-quark mass and some of the explicit $\mu _W$ dependence as well, see also [4] for more details. The parametric uncertainty due to the input value for the top-quark mass in Table 6 is denoted by “$m_t$”.

**Table 3:** The contribution in % of each operator to ${{\,\mathrm{Re}\,}}A_{0,2}$ and ${{\,\mathrm{Im}\,}}A_{0,2}$ at $\mu _{0,2}$.

	$Q_1$	$Q_2$	$Q_3$	$Q_4$	$Q_5$	$Q_6$	$Q_7$	$Q_8$	$Q_9$	$Q_{10}$
${{\,\mathrm{Re}\,}}A_0$	12.7	95.8	0.2	2.4	1.1	$-$12.0	0.1	$-$0.2	0.0	0.0
${{\,\mathrm{Re}\,}}A_2$	$-$27.4	128.6	0.0	0.0	0.0	0.0	0.3	$-$1.5	0.0	0.0
${{\,\mathrm{Im}\,}}A_0$	0.0	0.0	$-$2.7	$-$16.9	$-$7.5	121.8	$-$0.2	3.4	1.8	0.4
${{\,\mathrm{Im}\,}}A_2$	0.0	0.0	0.0	0.0	0.0	0.0	$-$5.8	120.2	$-$18.0	3.6

The various relative contributions of the operators to ${{\,\mathrm{Re}\,}}A_{0,2}$ and ${{\,\mathrm{Im}\,}}A_{0,2}$ are listed in Table 3 when using $\mu _{0,2}$. These numbers show that ${{\,\mathrm{Re}\,}}A_{0,2}$ are dominated by the current–current operators. In ${{\,\mathrm{Re}\,}}A_0$ the $Q_2$ dominates with almost 96%, whereas the $Q_1$ and $Q_6$ contributions of about 12% cancel each other and there are subleading $2\%$ and $1\%$ contributions from $Q_4$ and $Q_5$. In ${{\,\mathrm{Re}\,}}A_2$ the $Q_2$ of $129\%$ and the $Q_1$ of $27\%$ enter with opposite signs and there is a subleading contribution from $Q_8$ of $-\,1.5$%. On the other hand the ${{\,\mathrm{Im}\,}}A_0$ is dominated by QCDP operators, where the 121% contribution of $Q_6$ is mainly reduced by $Q_4$ and $Q_5$. The ${{\,\mathrm{Im}\,}}A_2$ is dominated by EWPs, in particular by $122\%$ due to $Q_8$, which is partially cancelled by $Q_9$. The $5\%$ corrections from $Q_7$ and $Q_{10}$ cancel each other.

In the SM $\varepsilon '/\varepsilon$ receives contributions from QCDP and EWP via the $I =0$ matrix elements and from EWP via the $I=2$ matrix elements, that exhibit quite some hierarchies as can be seen in (36) and (38), respectively. These hierarchies are strongly counteracted by those present in the Wilson coefficients $y_i$ at the two scales $\mu _0 = 4.006\,\text {GeV}$ and $\mu _2 = 3.0\,\text {GeV}$, where we evaluate ${{\,\mathrm{Im}\,}}\widetilde{A}_0$ and ${{\,\mathrm{Im}\,}}A_2$, respectively. This is illustrated by the following semi-analytic results of $\varepsilon '/\varepsilon$ that include the NNLO QCD corrections to EWPs [24]

25 $$\begin{aligned} \frac{\varepsilon '}{\varepsilon }= & {} {{\,\mathrm{Im}\,}}\lambda _t \cdot \Big \{ a (1 - \hat{\Omega }_\text {eff}) \big [ 8.12 \langle Q_3 \rangle _0 - 23.26 \langle Q_4 \rangle _0\nonumber \\&\quad + 5.47 \langle Q_5 \rangle _0 - 23.72 \langle Q_6 \rangle _0 \big ]\nonumber \\&\quad - 0.06 \langle Q_7 \rangle _0 + 0.25 \langle Q_8 \rangle _0 - 3.85 \langle Q_9 \rangle _0 + 0.66 \langle Q_{10} \rangle _0\nonumber \\&\quad + 1.42 \langle Q_7 \rangle _2 - 6.45 \langle Q_8 \rangle _2 + 70.33 \langle Q_9 \rangle _2 \Big \} . \end{aligned}$$

Here the experimental values of ${{\,\mathrm{Re}\,}}A_{0,2}$ have been used only in the denominator of (10). As a remark on the side, we note that at the scale $\mu _2 = 3\,\text {GeV}$ the relations $\langle Q_{3,4,5,6} \rangle _2 = 0$ hold because the RBC-UKQCD calculations so far do not include isospin-breaking corrections, neither due to quark masses nor quark charges. In consequence no such contributions appear in (25). Moreover isospin relations (37) have been used to substitute $\langle Q_{1,2,10} \rangle _2 \rightarrow \langle Q_9 \rangle _2$. As mentioned before in Sect. 2.4, a straight-forward application of the NLO QCD$\,\times \,$QED RG equations to the matrix elements to evolve them to some different scale will generate nonvanishing $\langle Q_{3,4,5,6} \rangle _2$, because the RG flow includes isospin-breaking effects from quark charges. The effect on $\varepsilon '/\varepsilon$ is discussed in further detail in Appendix C, where we provide the analogous result to (25) at $\mu = 1.3\,\text {GeV}$.

The hierarchy of the Wilson coefficients signaled for instance by large coefficients in front of $\langle Q_{3,4} \rangle _0$ is strongly counteracted by a hierarchy in the hadronic matrix elements modifying the pattern of the various contributions:

26 $$\begin{aligned} \begin{aligned} \frac{\varepsilon '}{\varepsilon } =&\, {{\,\mathrm{Im}\,}}\lambda _t \cdot \Big \{ a (1 - \hat{\Omega }_\text {eff}) \big [ - 0.57 \big |_{3,0} - 3.51 \big |_{4,0}\\&- 1.56 \big |_{5,0} +25.33 \big |_{6,0} \big ]\\&- 0.03 \big |_{7,0} + 0.70 \big |_{8,0} + 0.37 \big |_{9,0} + 0.07 \big |_{10,0}\\&+ 0.33 \big |_{7,2} - 6.91 \big |_{8,2} + 0.83 \big |_{9,2} \Big \}\,, \end{aligned} \end{aligned}$$

where the “$|_{i,I}$” indicate the origin of the contribution. This shows much clearer the relevance of $\langle Q_6 \rangle _0 \sim B_6^{(1/2)}$ and $\langle Q_8 \rangle _2 \sim B_8^{(3/2)}$ for $\varepsilon '/\varepsilon$ and to some extend $\langle Q_4 \rangle _0$. Eventually

27 $$\begin{aligned} \frac{\varepsilon '}{\varepsilon } =&{{\,\mathrm{Im}\,}}\lambda _t \cdot \Big \{ 19.69 \, a (1 - \hat{\Omega }_\text {eff}) \big |_{\text {QCDP},0} + 1.11 \big |_{\text {EWP},0}\nonumber \\&\quad - 5.75 \big |_{\text {EWP},2} \Big \} \end{aligned}$$

shows the contributions of QCDP in $I=0$ and the partial cancellation of EWP contributions from $I=0$ and $I=2$. Note that this statement is scale dependent, i.e. at some other scales $\mu _{0,2}$ the composition changes slightly due to RG flow.

The final result for $a = 1.017$, using $\hat{\Omega }_\text {eff}^{(8)} = 0.17 \pm 0.09$ in the octet scheme (12), with NNLO QCD in EWP and other parameters as collected in Tables 2 and  6 is

28 $$\begin{aligned} (\varepsilon '/\varepsilon )^{(8)}&= \left( 17.4 \pm 2.3 \big |^\text {ME}_\text {stat} \pm 4.9 \big |^\text {ME}_\text {syst} \pm 2.6 \big |_{\hat{\Omega }_\text {eff}}\right. \nonumber \\&\quad \left. \pm 1.0 \big |_{{{\,\mathrm{Im}\,}}\lambda _t} \; {}^{+0.2}_{-0.6} \big |_{\mu _c} \; {}^{+0.4}_{-0.6} \big |_{\mu _W}\pm 0.1 \big |_{m_t} \right) \times 10^{-4} \nonumber \\&= \left( 17.4 \pm 6.1 \right) \times 10^{-4}. \end{aligned}$$

Considering the new value $\hat{\Omega }_\text {eff}^{(9)} = 0.29 \pm 0.07$ from the nonet scheme (13) we find:

29 $$\begin{aligned} (\varepsilon '/\varepsilon )^{(9)}&= \left( 13.9 \pm 2.0 \big |^\text {ME}_\text {stat} \pm 4.2 \big |^\text {ME}_\text {syst} \pm 2.0 \big |_{\hat{\Omega }_\text {eff}}\right. \nonumber \\&\quad \left. \pm 0.8 \big |_{{{\,\mathrm{Im}\,}}\lambda _t} \; {}^{+0.2}_{-0.5} \big |_{\mu _c} \; {}^{+0.5}_{-0.7} \big |_{\mu _W} \pm 0.1 \big |_{m_t} \right) \times 10^{-4} \nonumber \\&= \left( 13.9 \pm 5.2 \right) \times 10^{-4}. \end{aligned}$$

There is a statistical error due to the matrix elements from the lattice, based on covariance matrices for $I=0$, propagated with Monte Carlo methods as well as individually available statistical errors for $I=2$ matrix elements. The systematic uncertainty due to various sources related to the lattice approach is entirely dominated by the $15.7\,\%$ systematic error of $\langle Q_6 \rangle _0$ in ${{\,\mathrm{Im}\,}}A_0$. The isospin-breaking corrections to QCDP from ChPT, summarized in $\hat{\Omega }_\text {eff}$ in (11), contribute a relative uncertainty of $15\,\%$. There is an overall relative uncertainty of $5.5\,\%$ from ${{\,\mathrm{Im}\,}}\lambda _t$ due to the CKM input.

The NNLO QCD corrections to EWPs lead to a decrease of $\varepsilon '/\varepsilon$ [4] and without them the central value would be $(\varepsilon '/\varepsilon )^{(8)}= 18.1 \times 10^{-4}$. Since our numerical input and the treatment of short-distance contributions differs slightly from RBC-UKQCD our central value does not agree exactly with their prediction $(\varepsilon '/\varepsilon )_\text {RBC-UKQCD} = 21.7\times 10^{-4}$ [21], such that after setting $a = 1.0$, $\hat{\Omega }_\text {eff}= 0.0$, and using only NLO QCD EWP we obtain slightly higher $\varepsilon '/\varepsilon = 22.6 \times 10^{-4}$, but well within the uncertainties. The inclusion of NNLO QCD EWP reduces this to $\varepsilon '/\varepsilon = 21.8 \times 10^{-4}$.

In our prediction we made only use of the experimental values ${{\,\mathrm{Re}\,}}A_{0,2}|_\text {exp}$ in the denominator of (10). As proposed in [9], in addition also in the numerator one of the $I=0$ and one of the $I=2$ matrix elements could be eliminated in favour of ${{\,\mathrm{Re}\,}}A_{0,2}|_\text {exp}$ to improve the accuracy in the framework of the SM. Here we did not adapt this strategy, because in agreement with the RBC-UKQCD collaboration [21], we did not find evidence for a substantial improvement when employing it to the $I=0$ amplitude. It must be also noted that this strategy leads to a slightly reduced value of ${{\,\mathrm{Im}\,}}A_0$ compared to the result without the additional information from ${{\,\mathrm{Re}\,}}A_0|_\text {exp}$.

The “$\Delta I = 1/2$ rule” is given by the ratio

30 $$\begin{aligned} \frac{{{\,\mathrm{Re}\,}}A_0}{{{\,\mathrm{Re}\,}}A_2}&= 20.0 {}^{+2.3}_{-2.1} \big |^\text {ME}_\text {stat} \pm 3.3 \big |^\text {ME}_\text {syst} \; {}^{+0.3}_{-0.2} \big |_{\mu _c} \; {}^{+1.4}_{-1.2} \big |_{\mu _W} \,, \end{aligned}$$

and agrees with the experimental result $22.35 \pm 0.05$. Our value almost coincides with the RBC-UKQCD prediction. The RBC-UKQCD lattice results show that QCD dynamics, present dominantly in current–current operators, is responsible for this large ratio thereby confirming the findings within DQCD obtained many years ago [11, 48]. This is also seen in another recent LQCD analysis [49].

## BSM master formula

In this section we report the updated master formula coefficients describing the new physics effects beyond the SM (BSM) in $\varepsilon '/\varepsilon$,

31 $$\begin{aligned} \frac{\varepsilon '}{\varepsilon }&= \left( \frac{\varepsilon '}{\varepsilon }\right) _\text {SM} + \;\; \left( \frac{\varepsilon '}{\varepsilon }\right) _\text {BSM}\,, \end{aligned}$$

which were first presented in [35, 36]. The BSM contribution to $\varepsilon '/\varepsilon$ is given by the weight factors $P_i$ for each Wilson coefficient $C_i(\mu _{\text {EW}})$ of the operators and their chirality-flipped counterparts listed in Table 4. The $P_i(\mu _{\text {EW}})$ contain the information of the RG evolution from the low-energy scale $\mu$ to the electroweak (EW) scale $\mu _{\text {EW}}$ and are linearly dependent on the hadronic matrix elements of the operators at the scale $\mu$, such that the $\mu$-dependence cancels. The master formula takes the simple form

32 $$\begin{aligned} \left( \frac{\varepsilon '}{\varepsilon }\right) _\text {BSM}&= \; \sum _i P_i(\mu _{\text {EW}}) {{\,\mathrm{Im}\,}}\Big [ C_i(\mu _{\text {EW}}) - C_i^\prime (\mu _{\text {EW}})\Big ] \,, \end{aligned}$$

with the $N_f=5$ effective Hamiltonian

33 $$\begin{aligned} \mathcal {H}^{(5)}_{\Delta S =1} = -\sum _i \frac{C_i(\mu _{\text {EW}})}{(1\,\, \text {TeV})^2} Q_i\,, \end{aligned}$$

leading to dimensionless Wilson coefficients and $P_i$ factors. The sum runs over all Wilson coefficients of the operators in Table 4. These operators are a complete basis for non-leptonic $\Delta S = 1$ transitions in the absence of any other light degrees of freedom [36]. The Wilson coefficients and their weight factors are evaluated at the particular value $\mu _{\text {EW}}= 160\,\text {GeV}$ of the EW scale. For more details we refer to [35, 36].

In Table 4 we summarize the updated $P_i$ factors after taking into account the most recent $I=0$ matrix elements reported by RBC-UKQCD [21]. Table 4 has been obtained by taking into account the tree-level matching [50] and one-loop running [51] below the EW scale using the public codes wilson [52] and WCxf [53]. Only the $P_i$ factors of operators in Class A are affected by this change, since they depend exclusively on matrix elements of the SM operators. In all other classes the $P_i$’s depend on matrix elements of BSM operators or the chromomagnetic operator $Q_{8g}$ and remain unchanged. The central values as well as statistical and systematic uncertainties of the $I=0,2$ matrix elements of all operators are listed in Table 5 at the common scale $\mu = 1.3 \,\text {GeV}$.

**Table 4:** Updated $P_i$ coefficients entering the master formula for NP effects in $\varepsilon '/\varepsilon$

Class	$Q_i$	$P_i$	$\frac{\Lambda }{\text {TeV}}$
A)	$Q_{VLL}^u = (\bar{s}^i \gamma _\mu P_L d^i)(\bar{u}^j \gamma ^\mu P_L u^j)$	$-3.3 \pm 0.8$	57
	$Q_{VLR}^u = (\bar{s}^i \gamma _\mu P_L d^i)(\bar{u}^j \gamma ^\mu P_R u^j)$	$-124 \pm 11$	351
	$\widetilde{Q}_{VLL}^u = (\bar{s}^i \gamma _\mu P_L d^j)(\bar{u}^j \gamma ^\mu P_L u^i)$	$1.1 \pm 1.2$	32
	$\widetilde{Q}_{VLR}^u = (\bar{s}^i \gamma _\mu P_L d^j)(\bar{u}^j \gamma ^\mu P_R u^i)$	$-430 \pm 40$	656
	$Q_{VLL}^d = (\bar{s}^i \gamma _\mu P_L d^i)(\bar{d}^j \gamma ^\mu P_L d^j)$	$1.8 \pm 0.5$	42
	$Q_{VLR}^d = (\bar{s}^i \gamma _\mu P_L d^i)(\bar{d}^j \gamma ^\mu P_R d^j)$	$117 \pm 11$	342
	$Q_{SLR}^d = (\bar{s}^i P_L d^i)(\bar{d}^j P_R d^j)$	$204 \pm 20$	451
	$Q_{VLL}^s = (\bar{s}^i \gamma _\mu P_L d^i)(\bar{s}^j \gamma ^\mu P_L s^j)$	$0.1 \pm 0.1$	7
	$Q_{VLR}^s = (\bar{s}^i \gamma _\mu P_L d^i)(\bar{s}^j \gamma ^\mu P_R s^j)$	$-0.17 \pm 0.04$	12
	$Q_{SLR}^s = (\bar{s}^i P_L d^i)(\bar{s}^j P_R s^j)$	$-0.4 \pm 0.1$	19
	$Q_{VLL}^c = (\bar{s}^i \gamma _\mu P_L d^i)(\bar{c}^j \gamma ^\mu P_L c^j)$	$0.5 \pm 0.1$	22
	$Q_{VLR}^c = (\bar{s}^i \gamma _\mu P_L d^i)(\bar{c}^j \gamma ^\mu P_R c^j)$	$0.8 \pm 0.1$	28
	$\widetilde{Q}_{VLL}^c = (\bar{s}^i \gamma _\mu P_L d^j)(\bar{c}^j \gamma ^\mu P_L c^i)$	$0.7 \pm 0.1$	26
	$\widetilde{Q}_{VLR}^c = (\bar{s}^i \gamma _\mu P_L d^j)(\bar{c}^j \gamma ^\mu P_R c^i)$	$1.3 \pm 0.2$	35
	$Q_{VLL}^b = (\bar{s}^i \gamma _\mu P_L d^i)(\bar{b}^j \gamma ^\mu P_L b^j)$	$-0.33 \pm 0.03$	18
	$Q_{VLR}^b = (\bar{s}^i \gamma _\mu P_L d^i)(\bar{b}^j \gamma ^\mu P_R b^j)$	$-0.22 \pm 0.03$	14
	$\widetilde{Q}_{VLL}^b = (\bar{s}^i \gamma _\mu P_L d^j)(\bar{b}^j \gamma ^\mu P_L b^i)$	$0.3 \pm 0.1$	17
	$\widetilde{Q}_{VLR}^b = (\bar{s}^i \gamma _\mu P_L d^j)(\bar{b}^j \gamma ^\mu P_R b^i)$	$0.4 \pm 0.1$	19
B)	$Q_{8g} \;\;\, = m_s (\bar{s} \sigma ^{\mu \nu } T^a P_L d)\, G^{a}_{\mu \nu }$	$-0.35 \pm 0.12$	18
	$Q_{SLL}^s = (\bar{s}^i P_L d^i)(\bar{s}^j P_L s^j)$	$0.05 \pm 0.02$	7
	$Q_{TLL}^s = (\bar{s}^i \sigma _{\mu \nu } P_L d^i)(\bar{s}^j \sigma ^{\mu \nu } P_L s^j)$	$-0.14 \pm 0.05$	12
	$Q_{SLL}^c = (\bar{s}^i P_L d^i)(\bar{c}^j P_L c^j)$	$-0.26 \pm 0.09$	16
	$Q_{TLL}^c = (\bar{s}^i \sigma _{\mu \nu } P_L d^i)(\bar{c}^j \sigma ^{\mu \nu } P_L c^j)$	$-0.15 \pm 0.05$	12
	$\widetilde{Q}_{SLL}^c = (\bar{s}^i P_L d^j)(\bar{c}^j P_L c^i)$	$-0.23 \pm 0.07$	15
	$\widetilde{Q}_{TLL}^c = (\bar{s}^i \sigma _{\mu \nu } P_L d^j)(\bar{c}^j \sigma ^{\mu \nu } P_L c^i)$	$-5.9 \pm 1.9$	76
	$Q_{SLL}^b = (\bar{s}^i P_L d^i)(\bar{b}^j P_L b^j)$	$-0.35 \pm 0.12$	18
	$Q_{TLL}^b = (\bar{s}^i \sigma _{\mu \nu } P_L d^i)(\bar{b}^j \sigma ^{\mu \nu } P_L b^j)$	$-0.11 \pm 0.03$	10
	$\widetilde{Q}_{SLL}^b = (\bar{s}^i P_L d^j)(\bar{b}^j P_L b^i)$	$-0.34 \pm 0.11$	18
	$\widetilde{Q}_{TLL}^b = (\bar{s}^i \sigma _{\mu \nu } P_L d^j)(\bar{b}^j \sigma ^{\mu \nu } P_L b^i)$	$-13.4 \pm 4.5$	115
C)	$Q_{SLL}^u = (\bar{s}^i P_L d^i)(\bar{u}^j P_L u^j)$	$74 \pm 16$	272
	$Q_{TLL}^u = (\bar{s}^i \sigma _{\mu \nu } P_L d^i)(\bar{u}^j \sigma ^{\mu \nu } P_L u^j)$	$-162 \pm 36$	402
	$\widetilde{Q}_{SLL}^u = (\bar{s}^i P_L d^j)(\bar{u}^j P_L u^i)$	$-15.6 \pm 3.3$	124
	$\widetilde{Q}_{TLL}^u = (\bar{s}^i \sigma _{\mu \nu } P_L d^j)(\bar{u}^j \sigma ^{\mu \nu } P_L u^i)$	$-509 \pm 108$	713
D)	$Q_{SLL}^d = (\bar{s}^i P_L d^i)(\bar{d}^j P_L d^j)$	$-87 \pm 16$	295
	$Q_{TLL}^d = (\bar{s}^i \sigma _{\mu \nu } P_L d^i)(\bar{d}^j \sigma ^{\mu \nu } P_L d^j)$	$191 \pm 35$	436
E)	$Q_{SLR}^u = (\bar{s}^i P_L d^i)(\bar{u}^j P_R u^j)$	$-266 \pm 21$	515
	$\widetilde{Q}_{SLR}^u = (\bar{s}^i P_L d^j)(\bar{u}^j P_R u^i)$	$-60 \pm 5$	244

The changes are moderate of not more than 30% for operators that contribute directly to $K\rightarrow \pi \pi$, whereas changes can be larger for those operators (with *s*, *c*, *b*-quarks) that enter via RG running from the EW scale down to the low-energy scale and have smaller coefficients. The last column of Table 4 shows the suppression scale $\Lambda$ that would generate $(\varepsilon '/\varepsilon )_\text {BSM} = 10^{-3}$ for $C_i = 1/\Lambda ^2$, assuming the presence of only this particular operator. For comparison, the theory uncertainty of the SM prediction (28) is about $0.6 \times 10^{-3}$. The scale $\Lambda$ is strongly dependent on the uncertainties of the matrix elements, which did not all decrease in the latest RBC-UKQCD predictions. A comparison to the previous values [35] shows a slight increase of $\Lambda$ for the first seven operators, which contribute directly to $K\rightarrow \pi \pi$. In general $\Lambda$ also increases for the remaining Class A operators, with a few exceptions, pushing the NP scale also in these cases up, even though they are entering only via RG mixing. This shows that the new results for the matrix elements from RBC-UKQCD will lead to stronger bounds on CP violation beyond the SM.

Eventually we point out that the large increase of the central value of $(\varepsilon '/\varepsilon )_\text {SM}$ in the SM from $\sim (1 - 2) \times 10^{-4}$ with the 2015 RBC-UKQCD results to $\sim 14 \times 10^{-4}$ with the 2020 results constitutes one order of magnitude and hence has significant impact on excluded regions of parameter spaces of BSM scenarios. The 2015 SM predictions [5, 9, 10] suggested a strong anomaly with a constructive $(\varepsilon '/\varepsilon )_\text {BSM} \approx (5-15) \times 10^{-4}$ to reach agreement with the experimental value $(\varepsilon '/\varepsilon )_\text {exp} = (16.6 \pm 2.3) \times 10^{-4}$. Contrary, the $(\varepsilon '/\varepsilon )_\text {SM}$ predictions based on 2020 results do not show anymore an anomaly, but allow now for both, a constructive and destructive interference, that can be still sizable in view of the large theory uncertainties

34 $$\begin{aligned} -4 \times 10^{-4} \;\lesssim \; \left( \frac{\varepsilon '}{\varepsilon } \right) _\text {BSM} \;\lesssim \; + 10 \times 10^{-4} \end{aligned}$$

as a rough $1\,\sigma$ range. The complete error propagation can be obtained properly for general BSM scenarios with the master formula, which is implemented in the public code flavio [54, 55]. Despite the large uncertainties, $\varepsilon '/\varepsilon$ was and remains one of the strongest constraints on CP violation in the quark-flavour sector, as has been shown for different BSM scenarios in the past. The BSM studies based on the 2015 SM predictions of $\varepsilon '/\varepsilon$ used mostly the working hypothesis of a constructive $(\varepsilon '/\varepsilon )_\text {BSM}$ of similar size, see references in [4], and the obtained conclusions for $0 < (\varepsilon '/\varepsilon )_\text {BSM}$ are still mostly valid.

## Summary and outlook

Our final result for $\varepsilon '/\varepsilon$ in (5) differs significantly from the one of the RBC-UKQCD collaboration but in view of large uncertainties in both results they are in agreement with each other and with experiment. But as emphasized in [23] the perfect agreement of (5) with the ChPT result in (3) is a pure numerical coincidence because the latter was obtained with $\hat{\Omega }^{(8)}_\mathrm{eff}$ in place of $\hat{\Omega }^{(9)}_\mathrm{eff}$ and with the values of $B_8^{(3/2)}\approx 0.55$ and ${{\,\mathrm{Im}\,}}\lambda _t\approx (1.35)\times 10^{-4}$, which differ from ours. Still it would be important to clarify whether the QCD dynamics enhancing the parameter $B_6^{(1/2)}$ over unity in LQCD and in ChPT is the same.

The recent advances in LQCD allow us to hope that in the coming years we should be able to have a value of $\varepsilon '/\varepsilon$ within the SM with a comparable error to the experimental one. In order to reach this goal and thereby to obtain an assessment on the allowed room for NP contributions to $\varepsilon '/\varepsilon$ it is important to perform a number of steps:

- A more precise determination of $\langle Q_6(\mu _0)\rangle _0$ or $B_6^{(1/2)}(\mu _0)$. At least a second LQCD collaboration should calculate $\varepsilon '/\varepsilon$, in order to confirm the large enhancement of $B_6^{(1/2)}$ found by RBC-UKQCD that has not been identified in DQCD. Also the errors in other matrix elements should be decreased.
- A more precise determination of $\hat{\Omega }_\text {eff}$. In particular in LQCD calculations isospin-breaking corrections and $\mathcal {O}(\alpha _{\text {em}})$ corrections in hadronic matrix elements required for the removal of renormalization scheme dependence at this order should be taken into account. The present status is summarized in [56].
- A more precise determination of the short distance contributions, especially in the QCD penguin sector, which in the context of the RBC-UKQCD analysis will decrease the sensitivity to the matching scale $\mu _c$. Despite the fact that the NNLO analysis of QCD corrections to EWP contributions practically removed the sensitivity of $\varepsilon '/\varepsilon$ to the renormalization scheme of the top-quark mass and $\mu _W$, our analysis shows that the significant $\mu _c$ uncertainty in the EWP sector still has to be removed through the matching of $N_f=4$ to $N_f=3$ effective theory at the NNLO level.
- The computation of the BSM $K\rightarrow \pi \pi$ hadronic matrix elements of four-quark operators by lattice QCD, which are presently known only from the DQCD approach [57].

Several BSM analyses of $\varepsilon '/\varepsilon$ have been performed, which are collected in [4]. A recent example of a $Z'$ model with explicit gauge anomaly cancellation has been discussed in [58]. Furthermore leptoquark models, except the $U_1$ model, would not be able to explain large deviations of the SM value from the data due to constraints coming from rare *K* decays [59]. This underlines the importance of correlations of $\varepsilon '/\varepsilon$ with other observables in NP scenarios. The new SM value in (5) removes the difficulties of leptoquark models pointed out in [59], but these problems could return with an improved analyses of $\varepsilon '/\varepsilon$ within the SM.

Furthermore the lessons from the SMEFT analysis in [36] should be useful in this respect. Such general analyses allow to take into account constraints from other processes such as collider processes, electroweak precision tests, neutral meson mixing as well as electric dipole moments. Finally the master formula for $\varepsilon '/\varepsilon$ presented in [35] valid for any BSM scenario should facilitate the derivation of constraints on CP-violating phases beyond the SM imposed by $\varepsilon '/\varepsilon$. In this respect we point out that also ${{\,\mathrm{Re}\,}}A_2$ has a very precise SM prediction and can be predicted rather precisely also in BSM scenarios, providing thus a second observable besides $\varepsilon '/\varepsilon$ to constrain also real parts of the Wilson coefficients of non-leptonic $\Delta S = 1$ operators.

## Data Availability

This manuscript has no associated data or the data will not be deposited. [Authors’ comment: No data was created during course of this project].

## Hadronic matrix elements

Here we collect the input for the $K\rightarrow (\pi \pi )_I$ isospin $I=0,2$ hadronic matrix elements

35 $$\begin{aligned} \langle Q_i \rangle _I&\equiv \langle (\pi \pi )_I | Q_i | K \rangle \end{aligned}$$

of the relevant operators in the traditional SM basis [20]. They are given for the $\overline{\text {MS}}$ scheme in order to combine them with the Wilson coefficients in Appendix B at the scale chosen by RBC-UKQCD. In addition we provide these matrix elements together with the complete set of non-leptonic $\Delta S = 1$ operators beyond the SM in Table 5 at the common scale $\mu = 1.3$ GeV. These results can be used for new physics studies using the master formula of $\varepsilon '/\varepsilon$ in [35, 36] that we updated in Section 4.

**Table 5:** Numerical values of $K\rightarrow \pi \pi$ hadronic matrix elements from the literature in units of $\text {GeV}^3$ in the $\overline{\text {MS}}$ scheme at the scale $\mu = 1.3\,\text {GeV}$, with statistical and systematic uncertainties. For the SM four-quark operators we provide the values obtained by RG equations with NLO QCD$\,\times \,$QED and only NLO QCD. The normalization convention is chosen to be $h=1$ for all operators.

$Q_i$	$\langle Q_i \rangle _0$		$\langle Q_i \rangle _2$	
SM operators				
	QCD$\,\times \,$QED	QCD	QCD$\,\times \,$QED	QCD
$Q_1$	$-$0.065(17)(10)	$-$0.065(17)(10)	0.0087(2)(5)	0.0085(2)(5)
$Q_2$	0.087(13)(14)	0.087(13)(14)	0.0085(2)(5)	0.0085(2)(5)
$Q_3$	$-$0.075(57)(12)	$-$0.075(57)(12)	0.0000	0
$Q_4$	0.093(51)(15)	0.093(52)(15)	$-$0.0003	0
$Q_5$	$-$0.120(53)(19)	$-$0.121(53)(19)	0.0002	0
$Q_6$	$-$0.641(46)(101)	$-$0.644(46)(101)	0.0011	0
$Q_7$	0.217(16)(34)	0.216(16)(34)	0.0996(68)(30)	0.0989(68)(30)
$Q_8$	1.583(30)(249)	1.581(30)(249)	0.684(19)(41)	0.683(19)(41)
$Q_9$	$-$0.059(17)(9)	$-$0.061(17)(9)	0.0132(3)(8)	0.0128(3)(8)
$Q_{10}$	0.092(18)(14)	0.092(18)(14)	0.0130(3)(8)	0.0128(3)(8)
$Q_{8g}$	$-$0.013(4)		0	0
Beyond the SM operators				
$Q_1^{\text {SLL},u}$	$-$0.005(1)		$-$0.0030(6)	
$Q_2^{\text {SLL},u}$	$-$0.044(9)		$-$0.031(6)	
$Q_3^{\text {SLL},u}$	$-$0.371(74)		$-$0.262(52)	
$Q_4^{\text {SLL},u}$	$-$0.214(43)		$-$0.151(30)	
$Q_1^{\text {SLL},d}$	0.0070(14)		$-$0.002(4)	
$Q_2^{\text {SLL},d}$	$-$0.088(18)		0.031(6)	
$Q_1^{\text {SLR},u}$	$-$0.015(3)		0.0030(6)	
$Q_2^{\text {SLR},u}$	$-$0.141(28)		0.050(10)	

The new results for $I=0$ matrix elements of the SM operators from the year 2020 are from the RBC-UKQCD lattice collaboration [21]. They are given at the scale $\mu _0 = 4.006\,\text {GeV}$ in the $N_f = 2 + 1$ flavour theory. As the current lattice calculation works in the isospin limit, out of the ten $\langle Q_{1\ldots 10} \rangle _0$ there are only seven linearly independent (for $h=1$):

36 $$\begin{aligned} \begin{aligned} \langle Q_1 \rangle _0&= -0.087(18)(14) ,\quad&\langle Q_2 \rangle _0 = +0.120(12)(19) , \\ \langle Q_3 \rangle _0&= -0.070(50)(11) ,\quad&\langle Q_5 \rangle _0 = -0.284(51)(45) , \\ \langle Q_6 \rangle _0&= -1.068(73)(168) ,\\ \langle Q_7 \rangle _0&= +0.628(19)(99) , \quad&\langle Q_8 \rangle _0 = +2.767(52)(434) . \end{aligned} \end{aligned}$$

The first and second errors are of statistical and systematic origin, respectively. In particular the statistical error comprises also a covariance matrix provided in [21] that we use for the uncertainty propagation in Table 5 and predictions of $\varepsilon '/\varepsilon$. The systematic uncertainty due to various sources in the lattice approach was estimated to be $15.7\,\%$ (see table XXV [21]) for each matrix element without providing correlations.

The $I = 2$ matrix elements of the SM operators are also from RBC-UKQCD [6] from the year 2015 for $N_f = 2 + 1$. In particular we use the results from the RI-SMOM  scheme (Table XVI) and convert them to the $\overline{\text {MS}}$ scheme with the scheme conversion factor Eq.$\,(66)$ in [60]. The matrix elements are given at $\mu _2 = 3\,\text {GeV}$ where they fulfill the isospin relations

37 $$\begin{aligned} \frac{3}{2} \langle Q_1 \rangle _2&= \frac{3}{2} \langle Q_2 \rangle _2 = \langle Q_9 \rangle _2\nonumber \\&= \langle Q_{10} \rangle _2, \langle Q_{3,4,5,6} \rangle _2 = 0 , \end{aligned}$$

and reduce to three independent ones (for $h=1$)

38 $$\begin{aligned} \langle Q_7 \rangle _2&= 0.2340(52)(70) , \quad \langle Q_8 \rangle _2 = 1.072(28)(64) , \nonumber \\ \langle Q_9 \rangle _2&= 0.0118(3)(7) , \end{aligned}$$

where we have increased the systematic uncertainty of the results of the RI-SMOM  by adding in quadrature the difference of the results in the RI-SMOM  and the RI-SMOM $(\gamma , \gamma )$ schemes as given in [6] to account for this additional source of systematic uncertainty. The RG-evolved results at $\mu = 1.3\,\text {GeV}$ are given in Table 5, see also [36].

The matrix elements of operators beyond the SM were calculated using DQCD in [57]. The single error is of parametric and systematic origin. The matrix element of the chromo-magnetic dipole operator $O_{8g}$ has been calculated in [61, 62] in 2017/18. Note that we use here the normalization of [35, 36].

## Wilson coefficients

Here we summarize the $\Delta S = 1$ Wilson coefficients at the various scales used in our analysis in the NDR-$\overline{\text {MS}}$ scheme using the NLO RG evolution from [20]. The numerical input entering the Wilson coefficients is fixed to values in Table 6. The central values for the threshold scales at which the top-, bottom and charm quark are subsequently decoupled are chosen as $\mu _W = m_W$ for $N_f = 6 \rightarrow 5$, $\mu _b = 4.2\,\text {GeV}$ for $N_f = 5 \rightarrow 4$ and $\mu _c = 1.3 \,\text {GeV}$ for $N_f = 4 \rightarrow 3$. We employ three-loop running of $\alpha _s$ including threshold quark mass effects such that $\alpha _s(\mu _c) = 0.3767$ and $1/\alpha _{\text {em}}(\mu _c) = 133.84$ in $N_f = 3$. For simplicity we use in the threshold corrections for $N_f = 5 \rightarrow 4$ for the bottom-quark mass the value $m_b = 4.2\,\text {GeV}$, which agrees very well with latest determinations of the $\overline{\text {MS}}$ result ${\overline{m}}_b({\overline{m}}_b) = 4.198 \,\text {GeV}$ [41]. For the charm-quark mass in the threshold corrections we use $m_c = 1.3\,\text {GeV}$, which is close to the $\overline{\text {MS}}$ result ${\overline{m}}_c({\overline{m}}_c) = 1.27 \,\text {GeV}$, when using ${\overline{m}}_c(3\,\text {GeV}) = 0.988 \,\text {GeV}$ [41]. We remind that the threshold corrections enter here for the first time at NLO, hence to be able to cancel some of the renormalization scheme dependences of the bottom- and charm-quark masses, one has to go to the NNLO order, as for example done in [25] in the case of QCD penguins.

**Table 6:** Numerical input for Wilson coefficients

Parameter	Value	Ref.	Parameter	Value	Ref.
$\alpha _s^{(5)}(m_Z)$	0.1181(11)	[39]	$m_Z$	91.1876 GeV	[39]
$\alpha _{\text {em}}^{(5)}(m_Z)$	1/127.955(10)	[39]	$m_W$	80.385 GeV	[39]
$s_W^2 = \sin ^2(\theta _W)$	0.23126	[39]	$m_t^\text {pole}$	173.1(6)(5) GeV	[39]

**Table 7:** The $\Delta S = 1$ Wilson coefficients at various scales $\mu$ in the NDR-$\overline{\text {MS}}$ scheme for the renormalization scales $\mu _W = \mu _t = m_W$, $\mu _b = 4.2\,\text {GeV}$ and $\mu _c = 1.3\,\text {GeV}$ using NLO and partial NNLO matching results for $y_{7,\ldots ,10}$. Further $y_{1,2} = 0$

	$\mu = 1.3\,\text {GeV}$		$\mu = 3.0\,\text {GeV}$		$\mu = 4.006\,\text {GeV}$	
	NLO	NNLO’	NLO	NNLO’	NLO	NNLO’
$z_1$	$-0.3938$	$\leftarrow$	$-0.2368$	$\leftarrow$	$-0.1984$	$\leftarrow$
$z_2$	1.2020	$\leftarrow$	1.1096	$\leftarrow$	1.0892	$\leftarrow$
$z_3 \times 10^2$	0.4231	$\leftarrow$	$-0.3540$	$\leftarrow$	$-0.4679$	$\leftarrow$
$z_4 \times 10^2$	$-1.2693$	$\leftarrow$	1.5289	$\leftarrow$	2.1423	$\leftarrow$
$z_5 \times 10^2$	0.4231	$\leftarrow$	$-0.3142$	$\leftarrow$	$-0.5236$	$\leftarrow$
$z_6 \times 10^2$	$-1.2693$	$\leftarrow$	1.0955	$\leftarrow$	1.5460	$\leftarrow$
$z_7 \times 10^4$	0.4780	$\leftarrow$	0.8969	$\leftarrow$	1.2560	$\leftarrow$
$z_8 \times 10^4$	0	0	$-0.9518$	$\leftarrow$	$-1.0783$	$\leftarrow$
$z_9 \times 10^4$	0.4780	$\leftarrow$	0.2914	$\leftarrow$	0.5490	$\leftarrow$
$z_{10} \times 10^4$	0	0	0.7362	$\leftarrow$	0.8552	$\leftarrow$
$y_3 \times 10^2$	2.6958	$\leftarrow$	2.0441	$\leftarrow$	1.8743	$\leftarrow$
$y_4 \times 10^2$	$-5.4542$	$\leftarrow$	$-5.3848$	$\leftarrow$	$-5.3689$	$\leftarrow$
$y_5 \times 10^2$	0.5579	$\leftarrow$	1.1474	$\leftarrow$	1.2634	$\leftarrow$
$y_6 \times 10^2$	$-8.2572$	$\leftarrow$	$-5.9125$	$\leftarrow$	$-5.4750$	$\leftarrow$
$y_7 \times 10^2$	$-0.0180$	$-0.0192$	$-0.0137$	$-0.0146$	$-0.0119$	$-0.0128$
$y_8 \times 10^2$	0.0981	0.1050	0.0622	0.0666	0.0547	0.0585
$y_9 \times 10^2$	$-1.1167$	$-0.9939$	$-1.0184$	$-0.9063$	$-0.9975$	$-0.8878$
$y_{10} \times 10^2$	0.3981	0.3025	0.2366	0.1798	0.1997	0.1518

The top quark mass $m_t(\mu _t)$ is in the $\overline{\text {MS}}$ scheme for $\mu _t = \mu _W$, obtained from the pole mass^6^ value given in Table 6: $m_t(m_t) = 163.5\,\text {GeV}$ and $m_t(\mu _W) = 173.2 \,\text {GeV}$. We follow [4] and include also important NNLO matching corrections [24] that resolve the NLO renormalization scheme ambiguities for our choice $\mu _t = \mu _W$ via the modifications of $y_{7, \ldots , 10}(\mu )$ at the low-energy scale of about 1.07, 1.07, 0.89 and 0.76 leading to the NNLO’ values in Table 7, which we adapt in the numerics. For further details we refer to [4]. The prime in this indicates that still small $\mathcal {O}(\alpha _W\alpha _s\sin ^2\theta _W)$ corrections are not included.

## Isospin-breaking RG effects

In this appendix we comment on isospin-breaking effects in the RG flow present in the anomalous dimension matrices that govern the scale dependence of Wilson coefficients and matrix elements of the operators. They are due to quark charges present in the definitions of the EWP operators and due to QED corrections, and are of purely perturbative origin known up to NLO in QCD$\,\times \,$QED [28, 29, 31]. To compute the Wilson coefficients at the low-energy scale in the $N_f = 3$ theory, the isospin-breaking effects in the RG flow are combined with initial Wilson coefficients, which contain further isospin-breaking effects of the SM at the electroweak scale. In the predictions of the amplitudes $A_{0,2}$ as well as for $\varepsilon '/\varepsilon$ the low-energy Wilson coefficients are multiplied by the corresponding matrix elements, which in principle leads to the cancellation of the renormalization scheme dependence.

The matrix elements contain the dynamics of QCD$\,\times \,$QED at scales below the low-energy scale, i.e. in the nonperturbative regime of QCD. In the calculation with lattice methods so far no isospin-breaking corrections have been included, i.e. a purely isospin-symmetric QCD setup is used by RBC-UKQCD that neglects effects due to different quark masses $m_u \ne m_d$ as well as QED effects^7^ due to different quark charges. For this reason, firstly, scheme dependences between Wilson coefficients and matrix elements can only cancel for the isosymmetric parts. Secondly, the initial values of the $I=2$ matrix elements fulfill the isospin relations (37) at the scale $\mu _2 = 3\,\text {GeV}$, where they have been calculated. These isospin relations become broken by the full QCD$\,\times \,$QED RG flow when evolving the matrix elements to a different scale, as can be seen for the example in Table 5 at $\mu = 1.3\,\text {GeV}$, as for example $3/2 \langle Q_1 \rangle _2 \ne 3/2 \langle Q_2 \rangle _2 \ne \langle Q_9 \rangle _2 \ne \langle Q_{10} \rangle _2$.

In particular nonvanishing $I=2$ matrix elements $\langle Q_{3,4,5,6} \rangle _2 \ne 0$ are generated at $\mu = 1.3\,\text {GeV}$ that enter $\varepsilon '/\varepsilon$ with large Wilson coefficients. At the scale $\mu = 1.3\,\text {GeV}$ the analogue result to (25) becomes

39 $$\begin{aligned} \begin{aligned} \frac{\varepsilon '}{\varepsilon }&= {{\,\mathrm{Im}\,}}\lambda _t \cdot \Big \{ a (1 - \hat{\Omega }_\text {eff}) \big [ 11.68 \langle Q_3 \rangle _0 - 23.63 \langle Q_4 \rangle _0\\&\quad + 2.42 \langle Q_5 \rangle _0 - 35.77 \langle Q_6 \rangle _0 \big ]\\&\quad - 0.08 \langle Q_7 \rangle _0 + 0.45 \langle Q_8 \rangle _0 - 4.31 \langle Q_9 \rangle _0 + 1.31 \langle Q_{10} \rangle _0\\&\quad - 260.96 \langle Q_3 \rangle _2 + 527.98 \langle Q_4 \rangle _2 - 54.00 \langle Q_5 \rangle _2\\&\quad + 799.32 \langle Q_6 \rangle _2 \\&\quad + 1.86 \langle Q_7 \rangle _2 - 10.16 \langle Q_8 \rangle _2\\&\quad + 96.21 \langle Q_9 \rangle _2 - 29.28 \langle Q_{10} \rangle _2 \Big \} . \end{aligned} \end{aligned}$$

The central values of the matrix elements in Table 5 lead then to $(\varepsilon '/\varepsilon )^{(8)} = 18.1\times 10^{-4}$ and $(\varepsilon '/\varepsilon )^{(9)} = 14.6\times 10^{-4}$, respectively. These results differ by about $+0.7\times 10^{-4}$ from the predictions (28) and (29), which are rather small variations in view of larger uncertainties in the prediction of $\varepsilon '/\varepsilon$. They might be viewed as an estimate of isospin-breaking corrections due to quark charges. However, the lacking contributions in the nonperturbative matrix elements could cancel them in large parts, as one would expect on the basis of renormalization scheme cancellations. We note that they are unrelated to isopin-breaking quark-mass effects, which are entirely of nonperturbative origin.

In the case of our preferred choice of calculating $\varepsilon '/\varepsilon$ at the scales $\mu _{0,2}$ where RBC-UKQCD has calculated $I=0,2$ matrix elements in the NDR-$\overline{\text {MS}}$ scheme, these isospin-breaking corrections are entirely contained in the Wilson coefficients. At a different scale $\mu$ one might impose the isospin relations for matrix elements by hand, but a more consistent possibility is the use of the isospin-conserving parts of the RG equation only, i.e. the NLO QCD evolution obtained by setting $\alpha _{\text {em}}= 0$. Then the $I=2$ matrix elements fulfill the isospin relations exactly, whereas for $I=0$ matrix elements they are slightly broken, because we have kept the small QCD mixing of EWP into QCDP operators. With this approach we obtain at $\mu = 1.3\,\text {GeV}$ the predictions $(\varepsilon '/\varepsilon )^{(8)} = 17.1 \times 10^{-4}$ and $(\varepsilon '/\varepsilon )^{(9)} = 13.6\times 10^{-4}$, respectively, differing by about $-0.3\times 10^{-4}$ from the predictions (28) and (29) at $\mu _{0,2}$. In particular we have used the NLO QCD evolution of matrix elements for the semi-analytic equation (20) and coefficients in Table 1.

In conclusion these studies show that nonvanishing $I=2$ matrix elements of QCDP operators due to isospin-breaking effects can have a non-negligible impact on $\varepsilon '/\varepsilon$. The size of such effects is completely unknown at present, contrary to isospin-breaking effects from $I=0$ matrix elements of QCDP operators, contained in $\hat{\Omega }_\text {eff}$. Here, the obtained variations are based on the residual scheme dependence from the isopin-breaking contributions due to quark charges in perturbation theory, contained in the anomalous dimensions. Their impact on $\varepsilon '/\varepsilon$ is numerically subleading compared to other, currently much larger uncertainties.

## Footnotes

1 See also [13, 17] for a critical analysis of this approach as used in the context of $\varepsilon '/\varepsilon$.

2 This has been demonstrated for the case of the BSM operators contributing to $K^0-\bar{K}^0$ mixing in [18].

3 See also the discussion in [23] on this point.

4 Note though that the used input for quark masses has been updated here, see Table 2. The associated uncertainties are not included, because in the expressions for $\varepsilon '/\varepsilon$ the dependence on these parameters cancels.

5 We use here $h=1$ as opposed to RBC-UKQCD collaboration that uses $h=\sqrt{3/2}$.
